# Base Excess and Beyond: Evolving Concepts in Acid-base Analysis

**DOI:** 10.4274/TJAR.2025.252127

**Published:** 2026-02-09

**Authors:** Özge Köner, Tuğhan Utku, Kubilay Demirağ, Levent Döşemeci

**Affiliations:** 1 Yeditepe University Faculty of Medicine, Department of Anesthesiology and Intensive Care, İstanbul, Türkiye; 2 Ege University Faculty of Medicine, Department of Anaesthesiology and Intensive Care, İzmir, Türkiye; 3 Memorial Antalya Hospital, Clinic of Anaesthesiology and Intensive Care, Antalya, Türkiye

**Keywords:** Acid-base equilibrium, alactic base excess, base excess, metabolic acidosis, metabolic alkalosis, Stewart approach

## Abstract

Base excess (BE), a marker used to detect metabolic acid-base disturbances, is also known to predict mortality in critically ill patients; the traditional concept, originally based on the Henderson-Hasselbalch model, has been further refined through integration with the Stewart approach, enabling a more comprehensive and mechanistic evaluation of acid-base disturbances. However, the increasingly complex mathematical formulations required for this integration demand extensive calculations, which can hinder bedside assessment. To address this, the BE formula has been simplified and integrated into the Stewart concept, resulting in a more reliable, detailed, and rapid bedside evaluation. Additionally, the term “alactic BE” was introduced to distinguish metabolic acidosis caused by retention of fixed acids from that caused by lactic acid accumulation, particularly in patients with renal failure. This review discusses the concept of BE and its evolution over the years.

**Main Points**

• The original concept of base excess (BE) was later expanded upon by the Fencl-Stewart model, which reframed the idea in terms of physicochemical principles.

• The Fencl-Stewart equations were rather intricate and impractical for quick calculations. To adress this, a simplified variation of the equation that maintains the core physicochemical concepts was introduced.

• The recent concept of “alactic BE” helps clinicians differentiate metabolic acidosis due to lactate accumulation from metabolic acidosis caused by retained fixed acids in patients with renal impairment.

• While the modern BE framework is more effective at identifying mixed acid-base disturbances, its clinical relevance is still uncertain.

## Introduction

Base excess (BE) is a fundamental parameter used to quantify metabolic acid-base disturbances and has long been recognized for its clinical utility. Beyond its diagnostic role, accumulating evidence indicates that BE also carries prognostic significance in critically ill populations.^1, 2, 3^In patients with acute kidney injury (AKI), both markedly low and high BE values on admission have been associated with an increased risk of 30-day mortality.^1^ Similarly, in critically ill patients with acute myocardial infarction, low BE has been identified as an independent predictor of short- and long-term mortality.^2^ A recent study in patients with ischemic stroke further demonstrated that a BE value below -3 mmol L^-1^ at intensive care unit (ICU) admission may indicate elevated mortality risk.^3^ Collectively, these findings highlight the potential of BE as a rapid and accessible early risk-stratification tool across a variety of acute and emergency conditions, including AKI, ischemic stroke, cardiac ischemia, and metabolic disturbances. Since its first description in 1948, the concept of BE has undergone substantial refinement and has been incorporated into contemporary approaches to acid-base interpretation, including the modern Stewart physicochemical framework. In this review, we examine the conceptual development of BE, its clinical applications, and its evolving role in modern acid-base evaluation.

### Buffer Base Concept

Buffer base (BB), which was introduced by Singer and Hastings^4^ in 1948, is the predecessor of the BE concept. According to this concept, plasma BB is practically equal to the sum of all buffer anions [total base (bicarbonate) and weak acid anions (e.g., albumin and phosphate)]. Therefore, BB increased either by an increase in base or by a decrease in plasma albumin concentration (Equation 1).

BB = HCO^-^_3_+A-                               (1)

The BB concept considers the non-carbonic buffers and is theoretically CO_2_-independent. Unfortunately, inter-subject variability due to differences in non-carbonic buffer concentrations was observed. To overcome this limitation, Siggaard-Andersen introduced the BE concept, in which the “excess” may be positive or negative, and which defines the actual BB relative to the normal BB (i.e., the actual BB minus the BB at normal pH and pCO_2_) as a measure of metabolic acid-base disturbance.^5, 6^

### Base Excess Concept

BE is the amount of acid or base (mmol L^-1^) that must be added to a blood sample to reach a pH of 7.40 inunder standardized conditions (pCO_2_ 40 mmHg, 37 °C).^6^ Affecters factors affecting of this semi-quantitative approach are pCO_2_ and BB, and; standard BE is used as its marker. BE is a Handerson Hasselbalch based Henderson-Hasselbalch- based parameter. It is calculated from the Van Slyke equation, which accounts for pH, HCO_3_ (mmol L^-1^) and haemoglobin (Hb, mmol L^-1^) (Equation 2).^7^

BE = (HCO^-^_3_−24.4+ [(2.3x Hb +7.7) x (pH-7.40)] + (1-0.023x Hb)                                                                       (2)

In this BE formula of Van Slyke, Hb can either be a constant value (16.2 mmol L^-1^) or be computed as a function of Hb concentration (assuming a constant protein concentration of 70 g L^-1^). The BE formula has also exhibits some inconsistencies due to changes in CO_2_. As in vivo BE is less reliable than in vitro BE, Van Slyke modified the equation to standardize the effect of Hb on BE [standard base excess (SBE)] and considered a lower Hb value for the calculation (Equation 3).

SBE = 0.9287 [HCO^-^_3_−24.4+14.83x (pH-7.40)]         (3)

However, the SBE equation assumes that the plasma albumin (g dL^-1^) and phosphate (mg dL^-1^) concentrations (Atot) are within the normal range. Therefore, Wooten described another formula for a multi-compartment model (Equation 4).^8, 9^ The normal value of SBE is between -2 and +2 mmol L^-1^.

Corrected SBE = (HCO^-^_3_−24.4) + [(8.3x albumin +0.15) + (0.29 x Pi x0.32)] x (pH-7.40)                                      (4)

**Figures 1:**
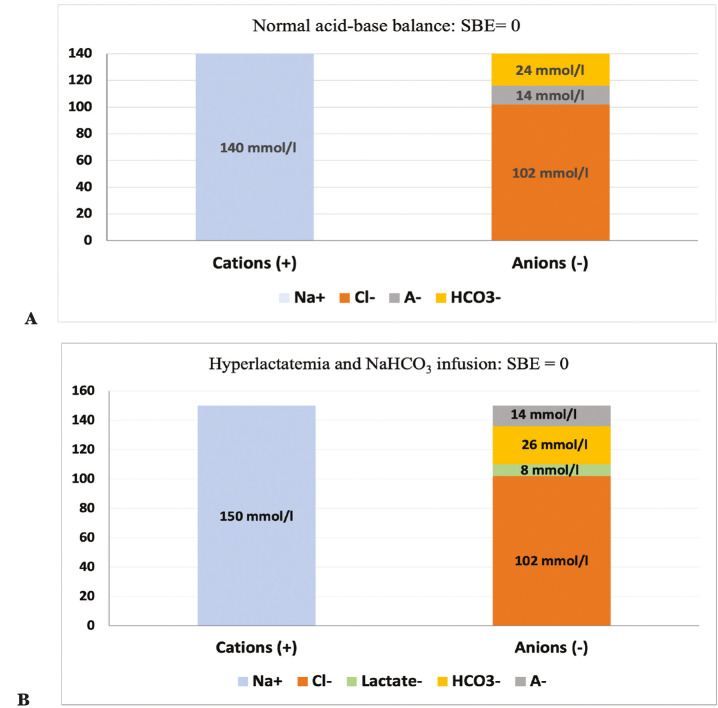
A, B. Gamblegrams show the standard base excess values of two patients, both are within the normal range (0). Figure 1 A represents a patient with normal acid-base balance, whereas Figure 1 B represents a patient with hyperlactatemia receiving a bicarbonate infusion. SBE, standard base excess; A-, non-volatile weak acids^12^

This multi-compartmental model has been shown to correlate well with the strong ion difference (SID) concept described by Stewart in that the change in SBE equals the change in SID across a vascular bed if there is no change in Atot.^8, 10, 11^ The anionic contribution of the most important weak acid, albumin (Atot), is 10 mEq L^-1^, whereas, unless phosphate levels are increased, their contribution to Atot is around 5%. If phosphate levels are elevated, then its contribution becomes more important. Because it is difficult to calculate Stewart’s SID equation, BE can be used as a substitute for SID at the bedside. Standard BE is a powerful concept in defining the metabolic acid-base disorders; however, it is not helpful for diagnosing the underlying pathology.^12^ Therefore, “mixed” acid-base disorders will go unnoticed without the use of detailed base-excess partitioning.^13, 14^ Furthermore, in some pathological situations, BE may be within the normal range and thus be misleading (Figure 1).^15^

Gamblegram^8, 16^ is a graphic representation of SID (therefore BE) developed by James Gamble (Figure 1). Assuming electroneutrality, the Gamblegram demonstrates that the ions that occupy the SID between the strong cations and anions are primarily bicarbonate (a component dependent on the SID) and the total concentration of weak acids.

Ciabattoni et al.^16^ compared Stewart’s SID and SBE approaches for detecting metabolic disturbances in a group of ICU patients receiving mechanical ventilation and found that the correlation between the two parameters was poor. They explained the results by stating that the SBE was derived from a variety of parameters, showed the effect of a single independent variable in the Stewart approach.^16^Stewart’s approach was superior to SBE in identifying metabolic alkalosis related to hypoalbuminemia and hypophosphatemia, and in identifying metabolic acidosis related to haemodilution following fluid resuscitation.

### Stewart Approach and its integration to BE concept

Unlike the classical Henderson-Hasselbalch approach, Stewart demonstrated that the concentration of [H^+^] in a physiological fluid is determined by three independent variables: the SID (which is equal to the difference between positively and negatively charged ions that are fully dissociated in biological fluids, e.g., Na^+^, K^+^, Cl^-^, lactate), the total concentration of weak acids (Atot), and the volatile acid CO_2_. According to Stewart, [H^+^] and [HCO_3-_] are dependent variables, and bicarbonate appears to be an indicator rather than a cause. Water becomes ionized under the influence of these independent variables, thereby regulating the pH. Stewart conducted his acid-base studies by applying the principles of electrical neutrality, the law of mass action, and conservation of mass. When expressed mathematically, apparent SID (SIDa) is equivalent to the “plasma BB” described by Singer and Hastings.^4^

### Fencl-Stewart Approach

To overcome the weaknesses of the SBE concept, Balasubramanyan et al.^17^ derived a new formula from the work of Fencl and defined it as the Fencl-Stewart approach. This derived formula combines BE with the Stewart approach. Gilfix et al.^18^ derived an equation to estimate the BE effects of the SID and the Atot. However, this formula is complex and difficult to apply at the bedside (Equation 5). Therefore, another equation, a simplified form of the Fencl-Stewart formula, was described by Story et al.^19^ (Equation 6).

BE = 0.3x [(Na^+^) -140] + 102-[(Cl^-^) x140/ (Na^+^)] + (0.123x pH- 0.631) x [42- albumin (g L^-1^)]                                                (5)

### Story’s Simplified Fencl-Stewart Approach

Rather than the complex formula of the aforementioned Fencl-Stewart approach, Story et al.^19^ proposed a simplified form (Equation 6). They confirmed in a study of 300 blood samples taken from critically ill patients that this simplified formula was in good agreement with the more complex Fencl-Stewart approach. Using this formula, the effects of SID and albumin on BE can be estimated.

SBE NaCl = {[Na^+^]-[Cl^-^]} -38 (38 is the average normal SID)                                                                                                  (6)

SBE Alb = 0.25× (42- measured albumin in blood)  (42 is the normal plasma albumin g L^-1^)

SBE NaCl + SBE Alb = SBE Correction

True SBE or BE gap = SBE - SBE Correction

The above equations require only basic mental arithmetic and are therefore easy to use at the bedside for evaluation, treatment, and follow-up of both simple and complex acid-base problems.

### Alactic Base Excess

Alactic BE (ABE) was defined by Gattinoni et al.^20^ to differentiate metabolic acidosis related to retention of non-lactate fixed acids (sulphates, phosphates) from that related to lactic acid in patients with renal failure (Equation 7).

ABE (mmol L^-1^) = SBE (mmol L^-1^) + Lactate (mmol L^-1^)    (7)

### Clinical and Research Consequences

### Studies with Fencl-Stewart approach

Traditional BE and bicarbonate methods can miss complex metabolic disorders in critically ill patients. While the Stewart and modified BE methods better identify multiple coexisting disturbances, their routine use may be more labor-intensive. The modified BE approach offers a practical balance between ease of use and clinical accuracy.^21^ In liver transplant patients, the Fencl-Stewart approach helps identify complex acid-base changes immediately after transplantation.^22^ Fencl-Stewart's method revealed frequent simultaneous metabolic acidosis and alkalosis missed by traditional analysis; both strong ion gap (SIG) and lactate independently predicted 28-day ICU mortality in critically ill.^23^

Balasubramanyan et al.^17^ found that unmeasured anions measure with Fencl-Stewart’s BE method correlates with mortality in pediatric intensive care unit patients, whereas Cusack et al.^24^ did not observe this in adults. To address the small sample sizes of earlier studies, Rocktaeschel et al.^25^ retrospectively analyzed 300 adult intensive care unit patients and found that unmeasured anions were the only acid-base variables with limited ability to predict mortality, while all calculation methods [anion gap (AG), corrected AG, SIG, and unmeasured anions measured by Fencl-Stewart BE] were strongly correlated with each other. Furthermore, of four methods for assessing chloride’s effect on acid–base status, chloride-specific base excess (BECl) most closely correlates with standard BE and most accurately reflects its impact in critically ill patients.^26^

### *A clinical example of simplified Fencl-Stewart approach by Story et al.* *^19^*

The patient underwent major gynaecological surgery with general anaesthesia. Isotonic sodium chloride was used as an intraoperative fluid. The metabolic acidosis, which developed 2 hours after induction of anaesthesia, is due to a decreased SID related to hyperchloremia. This effect was offset by a decrease in the concentration of albumin (a weak acid). There is no contribution from unmeasured ions in this case. This acidosis was observed after the infusion of 6 litres of isotonic sodium chloride (Table 1).

Na - Cl effect on BE = (Na^+^) - (Cl^-^) -38

Albumin effect on BE = 0.25x [42 - (Albumin) - g L^-1^]Unmeasured ion effect on BE = SBE - (Na^+^-Cl^-^) - Albumin effect

### *A clinical example comparing Fencl-Stewart, anion gap, and Stewart methods.* *^21^*

Table 2 presents a patient with postoperative multiple organ failure. The patient has complex acid-base physiology. The pH of 7.33, pCO_2_ of 30, and BE (-10) of -10 suggest metabolic acidosis and respiratory alkalosis; however, the etiology of the metabolic acidosis cannot be identified. By the AG method, the elevated corrected AG (ΔAGcorr =11 mEq L^-1^) suggests metabolic acidosis due to unmeasured anions. The delta-delta calculation predicts a bicarbonate level of 13-17 mEq L^-1^, and the observed bicarbonate of 15 mEq L^-1^ falls within this expected range. Based on this approach, no additional metabolic acid-base disorders are identified, even though a hypoalbuminemic alkalosis is intuitively suspected. In contrast, the Stewart approach reveals several abnormalities: an increased SIG, reduced sodium levels, elevated corrected chloride levels, and decreased albumin and phosphate levels. Together, these findings indicate a complex disturbance consisting of multiple metabolic acidoses (due to unmeasured anions, free-water excess, and hyperchloremia) and concurrent hypoalbuminemic and hypophosphatemic alkalosis. The modified BE method yields similar detail: a positive BEalb (+13 mEq L^-1^), a negative BECl (-8 mEq L^-1^), a negative BE for free water, and a negative BE for unmeasured anions (-8 mEq L^-1^), reflecting coexisting hypoalbuminemic alkalosis, hyperchloremic acidosis, dilutional acidosis, and unmeasured-anion acidosis. In this case, only the Stewart and modified BE methods accurately characterize all metabolic acid-base disturbances, whereas the AG method fails to detect the hypoalbuminemic alkalosis, hyperchloremic acidosis, and dilutional acidosis. The clinical significance of these missed abnormalities, however, remains uncertain.

### Alactic base excess

Gattinoni et al.^20^ studied ABE in septic patients and suggested that ABE may reflect the effects of renal dysfunction on plasma lactate concentration. Negative ABE values (<-3 mmol L^-1^) are correlated with mortality in patients with sepsis. Neutral ABE values (≥-3 to <4 mmol L^-1^) indicate that the kidneys effectively excrete fixed acids and maintain physiologic blood pH. Positive ABE values (≥4 mmol L^-1^) suggest that lactate and standard base levels are within the normal range in the blood, reflecting the kidney’s compensatory response to metabolic acidosis. In their study of intensive care patients with shock, Smuszkiewicz et al.^27^ reported that an admission ABE  below -3.63 mmol L^-1^ may be associated with an increased risk of 28-day all-cause mortality. Moreover, both BE and lactate levels—each contributing to ABE—offer independent and complementary predictive value.^27^ The combined criteria of BE -9.5 mmol L^-1^ and lactate >4.5 mmol L^-1^ identify patients at the greatest risk of death more effectively than BE, lactate, or ABE alone. Importantly, the prognostic significance of these metabolic markers remains robust regardless of patients’ age, sex, type of shock, or presence of severe renal failure. Negative ABE is also an independent predictor of in-hospital mortality among septic patients both with and without renal dysfunction, and among patients with acute myocardial infarction.^28, 29^

## Conclusion

In the mid-20^th^ century, the concept of BB, which represents the sum of the concentrations of bicarbonate, albumin, and haemoglobin, evolved into the concept of delta BB, defined as the change in BB from the “normal” value. Since the blood volume is diluted by interstitial fluid, the extracellular fluid model was introduced; this model uses a standard haemoglobin concentration (5 g dL^-1^ or 3 mmol L^-1^). Although it reflects only the non-respiratory (metabolic) component of acid-base disorders, the BE of the extracellular fluid remains valuable for etiological analysis, particularly when used alongside the Stewart approach in partitioning contributing factors. Clearly, the concept of BE continues to evolve, and ongoing refinements are likely. The Fencl-Stewart concept of BE has now been refined using the formula proposed by Story et al.^19^ This enables straightforward bedside calculations and helps identify the underlying pathology of metabolic acid-base disturbances, which the BE value alone cannot provide. The novel ABE concept further enhances our understanding by shedding light on the kidney’s role in metabolic acidosis.

## Figures and Tables

**Table 1. Simplified Fencl-Stewart approach.19:** 

**Measured values**	**After anaesthesia induction**	**2 hours after anaesthesia induction**
pH CO_2_ (mmHg) Na^+^ Cl^-^ Base excess (mEq L^-1^) Albumin (g dL^-1^) Na-Cl effect (mEq L^-1^) Albumin effect (mEq L^-1^) Effect of unmeasured anions (mEq L^-1^)	7.41 39.7 140 104 -0.4 4 -2 0.5 1.1	7.28 39.7 142 115 -6.7 2.8 -11 3.5 0.8

**Table 2. Comparing Fencl-Stewart, Anion Gap, and Stewart Methods.21:** 

**Measured values**	**Data**
pH CO_2_ (mmHg) Na^+ ^(mEq L^-1^) Cl^- ^(mEq L^-1^) Cl corrected (mEq L^-1^) K^+ ^(mEq L^-1^) BE lab (mEq L^-1^) Albumin (g dL^-1^) Pi (mmol L^-1^) HCO_3_ DAgap corrected Strong ion gap	7.33 30 117 104 112 3.9 -10 6 0.6 15 11 18
